# Anti-Enterovirus 71 Effects of Chrysin and Its Phosphate Ester

**DOI:** 10.1371/journal.pone.0089668

**Published:** 2014-03-05

**Authors:** Jianmin Wang, Ting Zhang, Jiang Du, Sheng Cui, Fan Yang, Qi Jin

**Affiliations:** MOH Key Laboratory of Systems Biology of Pathogens, Institute of Pathogen Biology, Chinese Academy of Medical Sciences and Peking Union Medical College, Beijing, People's Republic of China; University of North Carolina School of Medicine, United States of America

## Abstract

Enterovirus 71 (EV71) can cause severe disease and even lead to death in children, and an effective antiviral drug is currently unavailable. The anti-EV71 effect of chrysin (5,7-dihydroxyflavone), a natural flavonoid commonly found in many plants, was tested in this report. By using the predicting program Autodock 4.0 and an *in vitro* protease inhibition assay, we found that chrysin could suppress viral 3C^pro^ activity. Replication of viral RNA and production of viral capsid protein and the infectious virion were strongly inhibited by chrysin, without noticeable cytotoxicity. Cytopathic effects on cells were also prevented. Diisopropyl chrysin-7-yl phosphate (CPI), the phosphate ester for chrysin, was generated through a simplified Atheron-Todd reaction to achieve stronger anti-viral activity. CPI was also able to bind with and inhibit viral 3C^pro^ activity *in vitro*. As expected, CPI demonstrated more potent antiviral activity against EV71.

## Introduction

Enterovirus 71 (EV71) infection causes hand, foot, and mouth disease (HFMD), which is a common viral illness in infants and children. Most HFMD infections do not result in serious complications; however, some severe cases can present with serious neurological symptoms such as aseptic meningitis, encephalitis, and acute flaccid paralysis, and may even lead to death [1], [2], [3], [4]. Since the initial recognition in the United States, EV71 outbreaks have been observed frequently in Western Pacific Region countries [5], [6], [7], [8]. In 2008 alone, EV71 was confirmed to be responsible for the majority of the 488,955 infection cases in China, including 126 fatal cases [9]. Unfortunately, no effective drug or vaccine is clinically available to treat the disease. EV71 is a member of the *Picornaviridae* family. The viral genome is a ∼7.4-kb positive-sense, single-stranded RNA. There is a small viral protein, VPg, linked to the 5′ end of the genomic RNA instead of a 7-methyl guanosine cap. During infection, the virion is able to attach to the cellular Scavenger receptor B2 (SCARB2), P-selectin glycoprotein ligand-1 (PSGL-1) and heparin sulfate as cellular receptors [10], [11], [12]. After the virus invades the host cells, the viral genome will be translated into a polyprotein, which is cleaved in *cis* and in *trans* by virus-encoded proteases 2A^pro^, 3C^pro^, and 3CD^pro^ to produce approximately 10 final products as well as a number of cleavage intermediates [13], [14], [15], [16], [17]. In addition to its role in viral protein processing, the viral 3C protein also induces apoptosis and inhibits cellular polyadenylation [18], [19]. Recent studies have demonstrated that EV71 3C^pro^ can impair the antiviral response of the infected cell by disrupting retinoic acid-inducible gene I (RIG-I), Toll-like receptor 3 (TLR3), and interferon regulatory factor 7 (IRF7) signaling pathways [20], [21], [22]. Not only the crucial functions in viral replication, but also the lack of homologue of 3C^pro^ in human beings, results in the important role of 3C^pro^ in antiviral discovery.

Flavonoids are a broad class of more than 6,000 low-molecular-weight secondary metabolites that are present in all vascular plants. Flavonoid structure is usually characterized by a C_6_-C_3_-C_6_ carbon skeleton [23]. These phenolic compounds are often responsible for the bioactivities of plant crude extracts to afford protection against UV radiation, pathogens, and herbivores [24]. It has been reported that replication of a number of viruses, including herpes simplex viruses 1 and 2 (HSV-1 and HSV-2) and Japanese encephalitis virus (JEV), can be inhibited by baicalein, a member of the flavone subgroup of flavonoids [25], [26]. Quercetin, a member of the flavonol subgroup of flavonoids, is capable of inhibiting influenza virus, dengue virus, and JEV [26], [27], [28]. The antiviral activities of kaempferol and daidzin against JEV were also reported by our lab [29]. Because the structural information for EV71 3C^pro^ was characterized in our previous publication [15], the prediction of the 3C^pro^-ligand confirmation was performed using a predictive program, Autodock 4.0, to screen for the 3C protease inhibitor. Several flavonoids, including myricetin, daidzin, kaempferol and CR, were tested. However, only CR was predicted to bind with the 3C^pro^. The docking simulation result demonstrated that CR was able to interact with 3C^pro^ at several amino acid sites with negative binding energy (BE). We thus applied CR for in vitro protease inhibition assay and antiviral assay. In the *in vitro* protease inhibition assay, CR substantially inhibited EV71 3C^pro^ protease activity. In infected cells, CR strongly inhibited the synthesis of viral RNA and capsid protein, as well as the production of infectious virions. It also prevented cytopathic effects (CPE). Phosphorylated flavonoids demonstrated relatively stronger binding affinities and enhanced activity compared with non-phosphorylated forms [30], [31]. Therefore, we generated diisopropyl chrysin-7-yl phosphate (CPI), the phosphate ester for CR, through a simplified Atheron-Todd reaction. CPI was also able to bind with viral 3C^pro^ according to the docking simulation. Similarly, CPI inhibited 3C^pro^ activity *in vitro*. The antiviral activity of CPI against EV71 was also tested. As expected, CPI exhibited more potent antiviral activity against EV71.

## Results

### Inhibition of EV71 3C^pro^ activity *in vitro*

**Figure 1:**
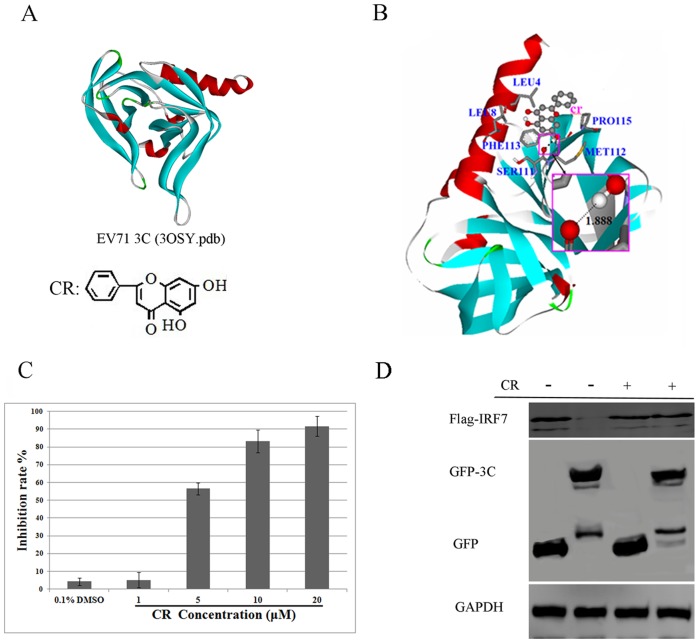
The inhibitory effect of CR against EV71 3C^pro^ *in vitro*. (A) The structure for EV71 3C^pro^ and the chemical structure for CR. (B) Molecular docking model of CR with viral 3C^pro^. (C) Protease activity of viral 3C^pro^ was inhibited by CR (*P*<0.05). Standard deviations of three independent experiments are shown.

Autodock 4.0 uses a semi-empirical free energy force field to predict the binding free energies of protein-ligand complexes in a known structure and binding energy for both bound and unbound states [32]. In this report we took advantage of Autodock 4.0 for stimulation and prediction of the 3C^pro^-CR confirmation. The structure of EV71 3C^pro^ and the chemical structure for CR was shown in Fig. 1A. Viral 3C^pro^-CR complexes were formed with CR stably posed in the pocket of 3C^pro^ in Autodock 4.0 (Fig. 1B). The binding sites were predicted as several hydrophobic and polar residues, including LEU4, LEU8, SER111, MET112, PHE113, and PRO115. A hydrogen bond was predicted to be formed between SER111 and the 7-OH of CR at around 2.0 Å. In addition to the binding site residue, the docking analysis also showed that the respective binding energy (BE) for 3C^pro^-CR was −5.55 kcal/mol. A negative BE value means that the complex is energetically stable; the more negative the BE is, the more stable the complex is. We performed a protease inhibition assay to test whether CR could inhibit 3C^pro^ activity *in vitro*. CR concentrations low as 10 µM substantially inhibited the protease activity of EV71 3C^pro^, with an IC_50_ of 4.03 µM (Fig. 1C). Our lab have previously found that EV71 3C protein led to cleavage of human interferon regulatory factor 7 (IRF7) in co-transfected 293T cells [22]. To determine whether CR could suppress protease activity for viral 3C^pro^ in cells, we co-transfected 293T cells with viral 3C and human IRF7 expressing plasmids in the presence or absence of 100 µM CR. 24 hours later, cells were harvested and the cleavage of IRF7 was detected by western blot. As shown in Fig. 1D, the cleavage of IRF7 by EV71 3C^pro^ was noticeably inhibited by CR. Taken together, computational analysis and the protease inhibition assay revealed that CR was able to suppress the protease activity of EV71 3C protein *in vitro* and in cells.

### Cellular toxicity of CR in RD cells

**Figure 2:**
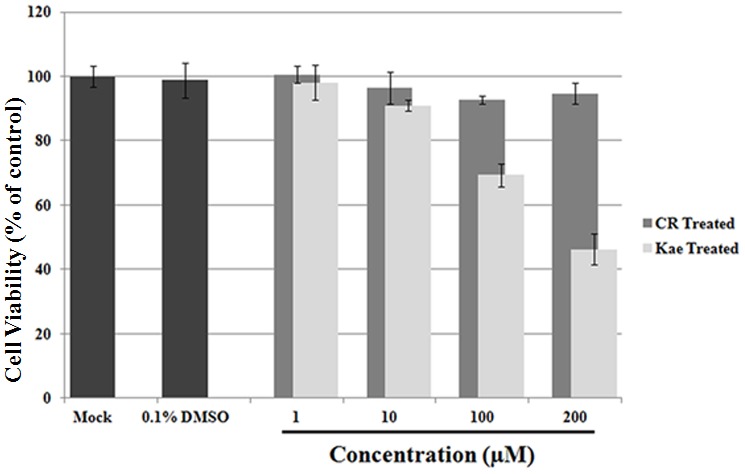
Assessment of the cytotoxicity of CR with respect to RD cells. 1–200 µM CR exhibited no obvious inhibitory effect on the proliferation of RD cells (*P*>0.5). The mean value was obtained from four replicate wells; means ± SD are shown.

Before applied into antiviral assay, we assessed the cellular toxicity of CR in RD cells using the MTS assay. When added to culture medium at concentrations of 1–200 µM for 72 h at 37°C, CR was tolerated by RD cells. No noticeable difference was detected in CR-treated cells compared with 0.1% DMSO-treated controls (Fig. 2). In contrast, Kae was cytotoxic to RD cells when added at concentrations exceeding 100 µM. CR has no detectable cytotoxicity to RD cells at the concentration of 200 µM.

### Inhibition of EV71 replication by CR

**Figure 3:**
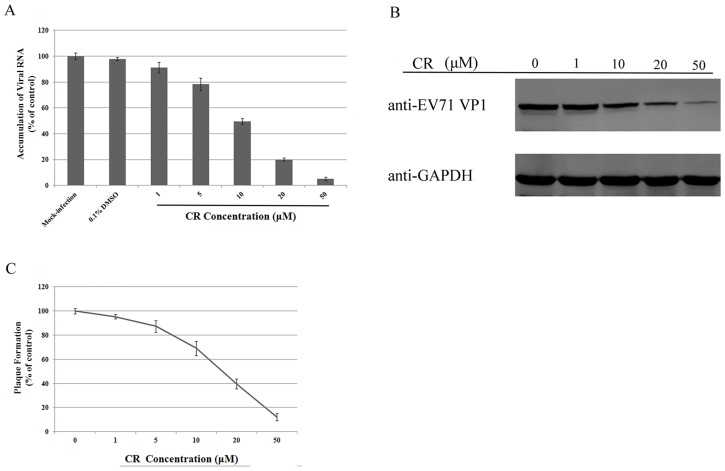
CR inhibits EV71 replication in RD cells. (A), (B) CR inhibited the accumulation of EV71 RNA and capsid protein VP1 synthesis in RD cells (*P*<0.05). (C) EV71 plaque formation was reduced by the addition of CR (*P*<0.05). The experiment was performed in triplicate; bars represent means ± SD.

To study the inhibitory effect of CR on EV71, RD cells were infected at 0.1 median tissue culture infective dose (TCID_50_) per cell, and the inhibitory effect of CR on viral RNA synthesis was measured at concentrations of 1, 5, 10, 20, and 50 µM. EV71 RNA accumulation in infected RD cells decreased as the concentration of CR increased (Fig. 3A). Ten micromolar CR inhibited EV71 replication by more than 50%, and less than 10% copy numbers for the EV71 genome were detected after the addition of 50 µM CR. CR inhibited EV71 RNA synthesis in a dose-dependent manner. The ability of CR to suppress replication of the EV71 RNA genome strongly suggested that overall synthesis of viral proteins would also be inhibited. Viral protein VP1 was detected by Western blotting. CR inhibited the synthesis of viral VP1 protein in EV71-infected cells in a dose-dependent manner (Fig. 3B). When the concentration of CR in the culture medium reached 20 µM, VP1 expression was dramatically reduced. In addition, CR inhibited EV71 plaque formation in a dose-dependent manner, with an IC_50_ of 24.12 µM (Fig. 3C).

### Protection of RD cells from EV71 infection

**Figure 4:**
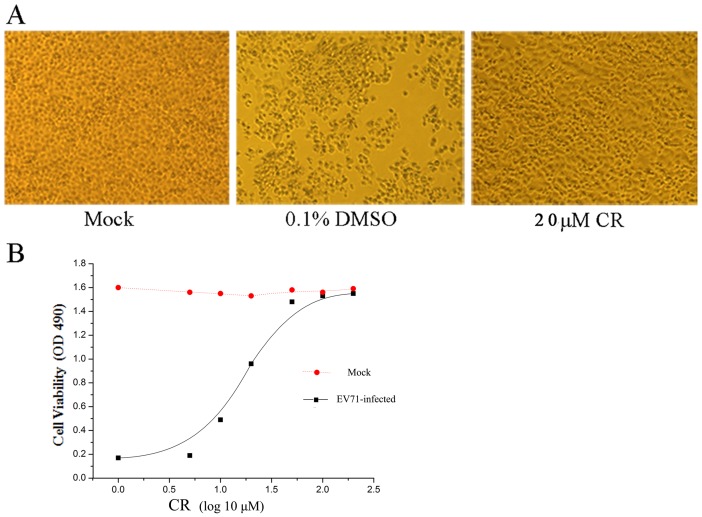
The effect of CR on EV71 infection. (A) CR reduced virus-induced cytopathic effects in RD cells. (B) CR protected RD cells from EV71 infection (*P*<0.05). The experiment was performed in triplicate; bars represent means ± SD.

To study the inhibition of EV71 CPE by CR, RD cells were infected at 0.1 TCID_50_/cell; at the time of infection, 20 µM CR was added to the culture medium. In the negative control experiment, cells were exposed to 0.1% DMSO which was used to dissolve the flavone. Morphological changes in the infected cells were examined using phase-contrast microscopy at 48 hpi. In contrast with the mock-treated control and the negative control, microscopy revealed that CR noticeably inhibited the CPE of EV71 on RD cells (Fig. 4A). EC_50_ value was also estimated by testing cell viability at 72 hpi through MTS assay. As shown in Fig. 4B, CR blocked EV71 proliferation with an EC_50_ of 15.89 µM.

### Inhibition of 3C activity by CPI

**Figure 5:**
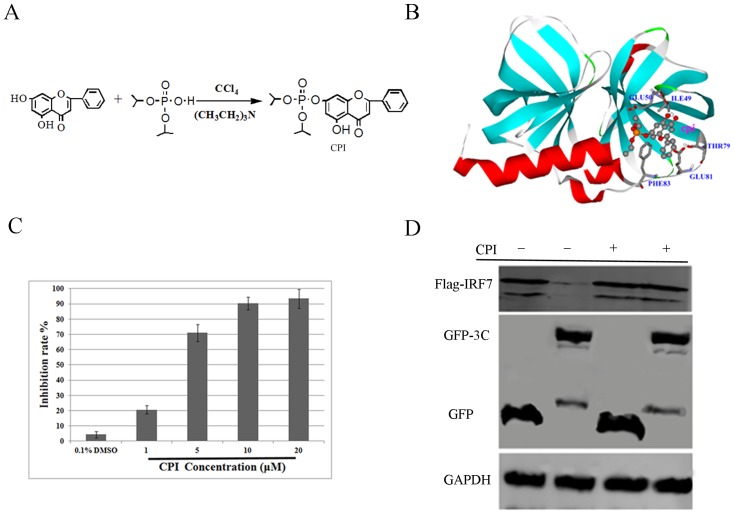
CPI inhibits EV71 3C^pro^ protease activity *in vitro*. (A) Synthesis of CPI using a simplified Atheron-Todd reaction. (B) Molecular docking model of CPI with viral 3C^pro^. (C) Protease activity for EV71 3C^pro^ was noticeably inhibited by CPI (*P*<0.05). Standard deviations of three independent experiments are shown.

We generated CPI, the phosphate ester of CR, through a simplified Atheron-Todd reaction (Fig. 5A). The structure of CPI was resolved and confirmed by NMR. We also analyzed the 3Cpro-CPI conformation using Autodock 4.0. CPI was predicted to bind with the same sites of EV71 3Cpro (Fig. 5B). With the presence of the diisopropyl phosphate group, CPI was predicted to form two H-bonds with PRO115 and VAL116 through the P-O bond at around 3.0 Å. At meanwhile, the corresponding BE was −6.09 kcal/mol, which was lower than the BE for 3Cpro-CR complexes. Next, we performed a protease inhibition assay to test the inhibitory effect of CPI on 3Cpro. CPI inhibited 3Cpro protease activity as potently as CR, with an IC50 of 1.9 µM (Fig. 5C). To further determine whether CPI could suppress protease activity for viral 3Cpro in cells, we co-transfected 293T cells with viral 3C and human IRF7 expressing plasmids in the presence or absence of 100 µM CPI. After 24 hours, cells were harvested and the cleavage of IRF7 was detected by western blot. As shown in Fig. 5D, the cleavage of IRF7 by EV71 3Cpro was noticeably inhibited by CPI.

### Antiviral activity of CPI

**Figure 6:**
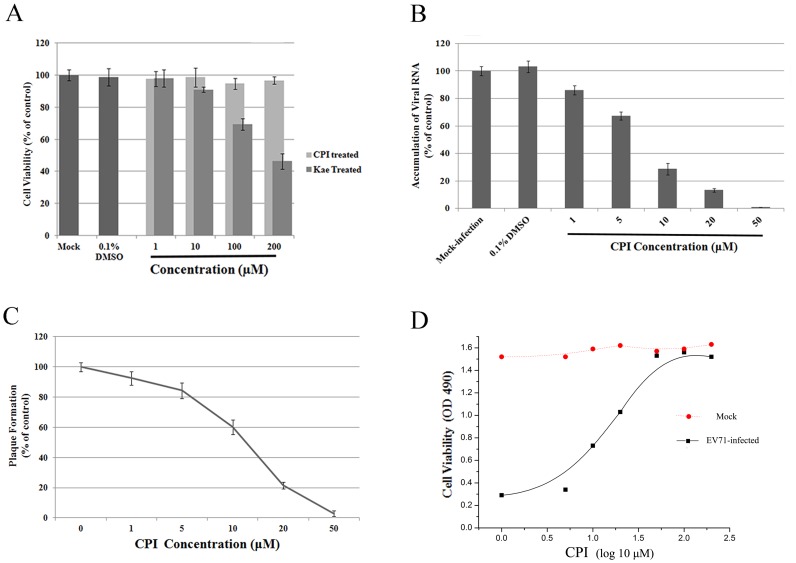
Anti-EV71 effect of CPI on RD cells. (A) 1–200 µM CPI exhibited no obvious inhibitory effect on the proliferation of RD cells (*P*>0.5). (B) EV71 RNA accumulation was substantially inhibited by CPI in RD cells (*P*<0.05). (C) CPI reduced plaque formation induced by EV71 infection (*P*<0.05). (D) CPI protected RD cells from EV71 infection. The mean value was obtained from three independent experiments; means ± SD are shown.

The cellular toxicity of CPI in RD cells was also assessed by using the MTS assay. No noticeable differences were detected in the CPI-treated group compared with 0.1% DMSO-treated controls when up to 200 µM CPI was added to the culture medium (Fig. 6A). Synthesis of viral RNA in the presence of CPI was also evaluated. CPI inhibited the replication of viral RNA (Fig. 6B). The production of EV71 induced plaque in the presence of CPI was also determined (Fig. 6C). CPI inhibited formation of EV71 plaque in a dose-dependent manner, with an IC_50_ of 13.86 µM, which is lower than that of CR. The inhibition of EV71 CPE by CPI was assessed. Complete protection against EV71-induced cell death was observed at the concentrations higher than 50 µM, with an EC_50_ of 9.06 µM (Fig. 6D).

### A CR/CPI-sensitive step after EV71 entry into cells

**Figure 7:**
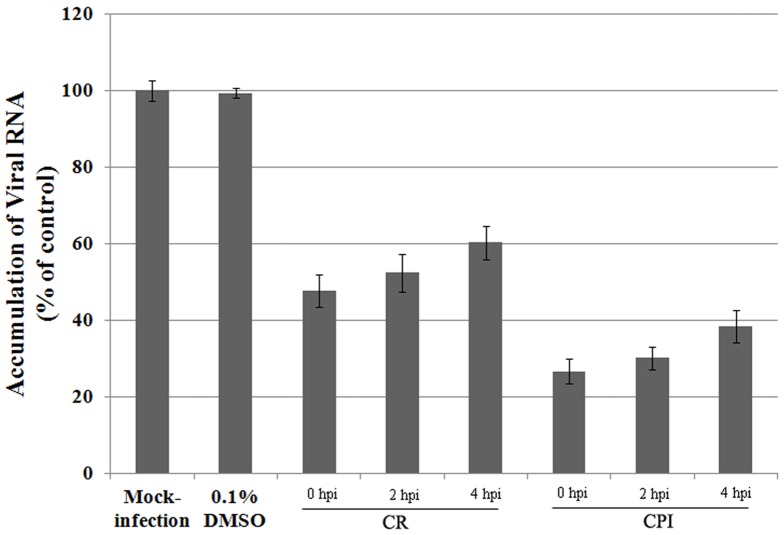
Effects of CR/CPI on established EV71 infection. CR/CPI, added 2 or 4 hpi, substantially inhibited EV71 replication in RD cells (*P*<0.05). Standard deviations of three independent experiments are shown.

The inhibitory effect of an anti-viral drug can affect any step in the infectious cycle, including cell entry, protein synthesis, RNA synthesis, or virion assemble and release. To determine whether the inhibitory effect of CR/CPI still occurred in an established EV71 infection, each of 10 µM CR/CPI was added to the culture medium up to 4 hpi. Replication of the EV71 RNA genome was noticeably inhibited by both compounds when added at 2 or 4 hpi (Fig. 7), and this effect was nearly as strong as when the drug was added at the time of infection, indicating that CR/CPI blocked EV71 replication by inhibiting the protease activity for viral 3C^pro^ after viral entry.

## Discussion

There is currently no effective antiviral drug available to treat patients infected by EV71. Intravenous immunoglobulin is usually used to treat severe cases, although its therapeutic efficacy seems limited [33]. Ribavirin [34], brefeldin A [35], aurintricarboxylic acid [36], and quinacrine [37] reportedly inhibit EV71 replication *in vitro*. However, none of these drugs can be used clinically ahead of sufficient clinical trials. In the current study, we found that CR and CPI from the flavone subgroup of flavonoid were highly effective against infectious EV71 replication in cell cultures, even at relatively low concentrations, without obvious cytotoxicity.

Flavonoids are secondary metabolites that are widely distributed in numerous plants, including flowers, fruits, and vegetables. Owing to relatively lower toxicity and a stronger bioactive potential to increase human health, there have been plenty of studies based on the development of pharmaceutical drugs. The structures of flavonoids are diverse and are determined by the number and arrangement of distinct substituents; however, the basic (C6-C3-C6) structure is present in all subgroups, including chalcones, flavones, flavanones, flavonols, and aurones. Several progressive investigations of the structure-activity relationship of flavonoids have been reported, including enzyme kinetics and molecular docking studies [38], [39].

With respect to the development of protein structural biology, automated docking is now extensively used as an effective means to quickly and accurately predict biomolecular conformations and BEs of protein-ligand complexes in molecular design [40]. Here, by using Autodock 4.0, we found that CR was likely to interact with 3C^pro^ at several amino acid sites, including LEU4, LEU8, SER111, MET112, PHE113, and PRO115, with a negative BE of −5.55 kcal/mol. Moreover, an *in vitro* protease inhibition assay revealed that CR inhibited the protease activity of EV71 3C^pro^, even at low concentrations. We also subjected CR to an antiviral test. As expected, EV71 replication was inhibited in infected RD cells. Since the diethyl phosphate esters of flavonoids possess relatively stronger binding affinities towards proteins such as myoglobin, insulin, and lysozyme and more easily form non-covalent compounds with them, compared to non-phosphorylated forms [30], [31]. In the study, we synthesized diisopropyl chrysin-7-yl phosphate (CPI) for more potent antiviral activity. NMR confirmed that CPI shares a similar structure with CR. The docking simulation indicated that, owing to the existence of the diisopropyl phosphate group, two H-bonds were formed through the P-O bond with EV71 3C^pro^. The BE for 3C^pro^-CPI complex was even lower (−6.09 kcal/mol). CPI also exhibited a more potent inhibitory effect against EV71 in infected cells. The IC_50_ for CPI was 13.86 µM, compared with 24.12 µM for CR. The life cycle of picornaviruses is rapid; virus entry, uncoating, and translation generally occur within 2 hpi, while viral RNA replication is initiated around 3 hpi. Here, we observed a CR/CPI-sensitive step after EV71 entry into cells. CR/CPI specifically reduced viral replication whether applied to cultured cells at the time of infection or four hours later. Plants, including Chinese herbal formulations, have been used to treat human diseases for centuries. The World Health Organization (WHO) estimates that approximately 80% of the global population still relies on traditional medicine for primary health care. The search for new bioactive molecules in plants is still an active part of pharmaceutical research in many key therapeutic areas, such as immunosuppression, infectious disease, oncology, and metabolic disease [41]. The screening of natural products has also led to the discovery of potent inhibitors of *in vitro* viral growth. Antiviral activities have been identified in several hundred natural compounds worldwide. There is no need of extra chemical synthesis to employ natural molecules derived from plant extracts versus pharmaceutical chemistry. This can reduce the cost of production, which is particularly pertinent to patient populations in low-income countries.

In conclusion, we have demonstrated that CR and CPI exert a strong inhibitory effect on EV71 replication. This finding is particularly important because no effective antiviral drug is currently available for the prevention, treatment, and control of EV71 infections in humans.

## Materials and Methods

### Test compounds

CR (5, 7-dihydroxyflavone, C_15_H_10_O_4_, Mr: 254.24) and kaempferol (Kae, C_15_H_10_O_6_, Mr: 286.23) were purchased from Sigma (St. Louis, MO), and CPI was synthesized using a simplified Atheron-Todd reaction. The new CPI compound was characterized by detailed spectroscopic analysis for C_21_H_23_O_7_P (MW: 418):^1^H NMR (400 MHz, DMSO) δ 12.752 (s, 1H), 7.915–7.894 (m, 2H), 7.566–7.543 (m, 2H), 7.271 (s, 1H), 7.015 (s, 1H), 6.673 (s, 1H), 6.668 (s, 1H), 4.812 (q, *J* = 6.4 Hz, 2H), 1.394 ppm (dd, *J* = 11.6, 6.0 Hz, 12H); ^31^P NMR (400 MHz, D_2_O), d: −9.073; ESI-MS/MS, m/z 419 [M+H]^+^, 391[M-C_2_H_4_+H]^+^. For cell experiments, the compounds were dissolved in dimethyl sulfoxide (DMSO) and then diluted in culture medium. To avoid toxicity or interference of the solvent, the maximum concentration of DMSO in the cell test medium was less than 0.1%.

### Cell culture

Rhabdomyosarcoma cells (RD; ATCC, Manassas, VA) were propagated and maintained in minimum essential medium (HyClone, Logan, UT) supplemented with 10% fetal bovine serum (Invitrogen, Carlsbad, CA) plus 100 U/ml penicillin and 100 µg/ml streptomycin at 37°C with 5% CO_2_. Cell numbers and proliferation were determined by direct cell number counting using CountStar (Inno-Alliance Biotech, Beijing, China) after staining with trypan blue.

### Viral infection and drug treatment

The EV71 strain (Shzh-98, GenBank accession no. AF302996) was used in all assays in this study. Viruses were propagated in RD cells. CR and CPI were added to cell cultures in a concentration series at the time of infection or at the indicated times post-infection. Western blotting and quantitative real-time PCR were performed 24 h post-infection (hpi) as described below. Virus titer was determined as TCID_50_ on RD cells using the Reed-Muench method [42]. Representative results are shown.

### MTS assay

Cellular toxicity for the tested compounds was evaluated using a previously reported cell viability assay [43]. The cells were harvested during the log phase of growth and inoculated onto 96-well plates at a final concentration of 3×10^3^ cells per well. After 24 h of incubation at 37°C under 5% CO_2_, the cell cultures were treated with flavones at concentrations of 1, 10, 100, and 200 µM. Twenty microliters of 3-(4,5-dimethylthiazol-2-yl)-5-(3-carboxymethoxyphenyl)-2(4-sulfophenyl)-2H-tetrazolium/phenazine methosulfate (MTS/PMS; Promega, Madison, WI) were added to each well, and the absorbance at 490 nm was measured according to the manufacturer's recommendations. The mean value was obtained from four replicate wells. A control group treated with 0.1% DMSO was evaluated simultaneously.

### Quantitative real-time PCR

At 24 hpi, total cellular RNA and viral RNA were extracted from each well using the RNAeasy Mini kit (Qiagen, Hilden, Germany) according to the manufacturer's instructions, and reverse transcribed using the Superscript First-Strand Synthesis System (Invitrogen, Carlsbad, CA) in a 20-µl reaction mixture with 1.2 µg total RNA and 50 ng random hexamers according to the manufacturer's protocol. Quantitative real-time PCR was conducted using an ABI Prism 7000 Real-time PCR System (Applied Biosystems, Carlsbad, CA) and the Power SYBR Green PCR Master Kit (Invitrogen, Carlsbad, CA). Nucleotides 1–750 in the 5′ untranslated region of the EV71 strain Shzh-98 genome sequence were amplified by PCR and cloned into a pGEM-T Easy vector (Promega, Fitchburg, WI) to construct the standard plasmid. Using the standard plasmid, we obtained the standard curve. Reactions contained 2 µl of cDNA, 1 µl of each primer, and 25 µl of Power SYBR Green PCR Master Mix in total volume of 50 µl. To control for equal loading, cellular glyceraldehyde-3-phosphatase dehydrogenase (GAPDH) was also assayed as an internal control. The following primers were used: for EV71, primers qEV-F (5′-CCCCTGAATGCGGCTAAT-3′) and qEV-R (5′-CAATTGTCACCATAAGCAGCCA-3′); for GAPDH, primers qGAPDH-F (5′-CTCTGCTCCTCCTGTTCGAC-3′) and qGAPDH-R (5′-TTAAAAGCAGCCCTGGTGAC-3′).

### Western blotting

Western blotting assays were conducted as below. First, cells were collected and washed with PBS twice before lysing in buffer containing 100 mM NaCl, 20 mM Tris (pH 8.0), 0.5% NP-40, 0.25% sodium deoxycholate, 1 mM EDTA with protease inhibitor cocktail. Proteins from the supernatant were then collected at 15,000 g for 15 min, resolved by electrophoresis in denaturing 4% to 10% SDS-PAGE, and transferred to nylon polyvinylidene difluoride (PVDF) membranes (Hybond P, Piscataway, NJ). Membranes were blocked with 5% nonfat dry milk and probed with primary antibodies as indicated at 4°C overnight, followed by incubation with the corresponding IRD Fluor 680-labeled IgG secondary antibody (Li-Cor Inc., Lincoln, NE). After washing, membranes were scanned using an Odyssey Infrared Imaging System (Li-Cor, Lincoln, NE) at the recommended wavelength and analyzed using Odyssey software. EV71 capsid protein VP1 was detected using anti-EV71 VP1 monoclonal antibody (eENZYME, Montgomery Village, MD). GFP-3C and Flag-IRF7 was detected by using mouse anti-GFP and anti-Flag (Beyotime, Suzhou, China). To control for protein loading, levels of the housekeeping protein GAPDH were assessed using mouse anti-GAPDH primary antibody (Beyotime, Suzhou, China) and IRD Fluor 680-labeled IgG secondary antibody (Li-Cor Inc., Lincoln, NE). The molecular sizes of proteins were determined by comparison with prestained protein markers (Fermentas, Glen Burnie, MD).

### Plaque reduction assay

Approximately, 10^6^ RD cells/well in 6-well plates were inoculated and incubated for 24 h to form a monolayer. Cells were then infected with EV71 (100 PFU/well) in the absence and presence of the serially diluted testing compounds. After 1 h incubation at 37°C, confluent layers of RD cells were covered with the overlay medium containing 1.5% agar and the testing compounds at the corresponding concentrations and incubated in a CO_2_ incubator for 3 days at 37°C. Finally, cell layers were fixed with 10% formaldehyde and stained with 0.05% crystal violet. The concentration necessary to reduce the number of plaques by 50% (50% inhibitory concentration, IC_50_) was then determined.

### Molecular docking simulation

To estimate the potential interaction and the conformation of the 3C^pro^-ligand complex, the flavone ligands were docked into the EV71 3C protein using the AutoDock 4.0 program (Scripps Research Institute, La Jolla, CA). The 3D structures for CR and CPI were constructed and minimized using Chemsketch 3.5 and Omega 2.0 software (OpenEye Scientific Software, Santa Fe, NM). For docking studies, we used the crystal structure of the EV71 3C^pro^ (PDB no. 3OSY). The predicted protein-flavone complexes were optimized and ranked according to the empirical scoring function, ScreenScore, which estimated the binding free energy of the ligand receptor complex. Docking of the 3C^pro^-flavone molecule was successful, as indicated by statistically significant scores. Each docking was performed twice, and each operation screened 250 conformations for the protein-ligand complex that were advantageous for docking; each docking had 500 preferred conformations. The most stable conformation as distinguished by the minimal binding energy was shown.

### Protease inhibition assay

To evaluate the inhibition of EV71 3C^pro^ protease activity by each flavone compound, we conducted a protease inhibition assay similar to a previously described method [44]. The fluorogenic peptide Dabcyl-RTATVQGPSLDFE-Edans (EV71 3C^pro^ cleaves the peptide bond between the underlined residues), corresponding to the EV71 polyprotein 3B/3C autoprocessing site, was used as the substrate. Recombinant EV71 3C protease was produced by prokaryotic expression as described previously [15] and used as an enzyme in the protease assays. Viral 3C^pro^ was first incubated with each flavone for 30 min at 4°C to allow sufficient binding. Next, the reactions were performed with 4 µM protease and 150 µM fluorogenic peptide in a buffer of Tris-HCl (pH 7.0), 200 mM NaCl, and 2 mM dithiothreitol (DTT) at 30°C. The fluorescence change resulting from the reaction was monitored using a fluorescence plate reader (Fluoroskan Ascent from Thermo Labsystems, Finland) at 538 nm with excitation at 355 nm. Enhanced fluorescence due to cleavage of the peptide was detected. For IC_50_ measurements, the initial velocities of the proteolysis were plotted against the different inhibitor concentrations by fitting with the equation: *A*(*I*) = *A*(0)×{1−([*I*])/([*I*]+IC_50)_]}, in which *A*(*I*) is the enzyme activity with corresponding inhibitor at the concentration of [*I*] and *A*(0) is enzyme activity without inhibitor, as described previously [44].

### Statistical analysis

At least three independent experiments were carried out for each variable. Statistical significance was assessed with SSPS version 10.0. Differences were considered statistically significant at a threshold of *p<0.05*. All results are presented as mean ± SD.
